# Choroidal features in flat irregular pigment epithelial detachment associated with Chronic central serous chorioretinopathy: Avascular versus vascularized

**DOI:** 10.1371/journal.pone.0257763

**Published:** 2021-09-23

**Authors:** Hooshang Faghihi, Alireza Mahmoudi, Elias Khalili Pour, Nazanin Ebrahimiadib, Kaveh Fadakar, Fariba Ghassemi, Ahmad Mirshahi, Alireza Khodabande, Hassan Khojasteh, Fatemeh Bazvand, Mohammadreza Mehrabi Bahar, Hamid Riazi-Esfahani

**Affiliations:** Retina Service, Farabi Eye Hospital, Tehran University of Medical Sciences, Tehran, Iran; University of Nebraska Medical Center, UNITED STATES

## Abstract

**Purpose:**

To investigate the differences in the choroidal biomarkers between two forms of flat irregular pigment epithelial detachment (FIPED): avascular (aFIPED) and vascularized (vFIPED) in eyes with chronic central serous chorioretinopathy (CSC).

**Materials and methods:**

Enhanced depth imaging optical coherence tomography (EDI-OCT) was done in eyes with FIPED correlated to chronic CSC, fellow eyes, and also in healthy eyes from gender- and age-matched subjects. Eyes with FIPED were classified into two subgroups based on optical coherence tomography angiography (OCTA) findings: vFIPED and aFIPED. Different choroidal biomarkers such as subfoveal choroidal thickness (SFCT), total choroidal area (TCA), and choroidal vascular index (CVI) were compared between the groups.

**Results:**

Forty-four eyes from 42 patients with chronic CSC and FIPED along with 40 eyes from 20 healthy subjects were included. OCTA identified vascularization in 14 eyes in the FIPED group (31.8%). Mean SFCT was higher in the FIPED group compared to two other groups (p = 0.005). In comparison to patients with aFIPED, patients with vFIPED had lower SFCT (p = 0.003) and higher CVI (p = 0.020) based on multivariate analysis.

**Conclusions:**

It seems that measurement of CVI along with SFCT may help to differentiate aFIPED from vFIPED in patients with CSC. Further longitudinal studies would be required to confirm the clinical significance of these findings.

## Introduction

The hallmark of the pachychoroid spectrum is choroidal vascular congestion and hyperpermeability which may be followed by focal changes in retinal pigment epithelium (RPE) such as pigment epithelial detachment (PED) [1].

In the continuum of pachychoroid spectrum, central serous chorioretinopathy (CSC) can occur as serous detachment of the neurosensory retina which is frequently accompanied by PED [1,2]. PED happens in 70% to 100% of eyes with CSC and can appear as a dome-shaped serous type in acute CSC or flat irregular type in chronic CSC [3].

The incidence and features of flat irregular pigment epithelial detachment (FIPED) in chronic CSC have been explored in few studies. However, thanks to the expanded use of multimodal imaging, it has been shown that high accordance exists between the location of the choroidal vascular abnormality and the region of FIPED formation. It has also been reported that these FIPEDs are strongly associated with type I choroidal neovascularization (CNV) [4,5].

In the present study, we evaluated the clinical characteristics and choroidal biomarkers of FIPED in eyes with chronic CSC and compared these features between avascular FIPED (aFIPED) and those which progressed to become vascularized (vFIPED).

## Materials and methods

This case-control study included treatment naïve patients with chronic CSC who had FIPED, referred to the Retina clinic of Farabi eye hospital, Tehran University of Medical Sciences, Tehran, Iran, between March 2019 and July 2020. The study protocol adhered to the principles outlined in the Declaration of Helsinki and was approved by the Tehran University of Medical science’s Institutional Review Board. Informed consent was obtained from each participant before inclusion in this observational study.

The presence of serous macular detachment associated with pachychoroid (detected with Enhanced-depth imaging optical coherence tomography (EDI-OCT)) and choroidal vascular hyperpermeability revealed on indocyanine green angiography led to the diagnosis of CSCR. Chronic CSCR was defined as the presence of symptoms for at least 6 months [6]. Eyes were enrolled in the study if they had macular FIPEDs on structural optical coherence tomography (OCT) cross-sectional B-scans. An FIPED was an irregular elevation of the RPE under which the Bruch’s membrane (BM) could be evident on OCT B-scans [7].

Eyes with refractive error (spherical equivalent) ≥ ±3 diopters, any sign of age-related macular degeneration (ARMD), history of glaucoma, intraocular inflammation, prior photodynamic therapy (PDT), intravitreal anti-VEGF injection, laser photocoagulation, and those with poor quality EDI-OCT images were excluded. To maintain a consistent phenotype, patients who had a pachychoroid but no history of serous retinal detachment, or those who had polypoidal choroidal vasculopathy revealed by dye angiography were excluded. Patients with systemic conditions that may affect hemodynamic regulation such as systemic hypertension (systolic BP >150 mm Hg or diastolic BP >90 mm Hg) or diabetes were also excluded from the study.

Eyes with chronic CSC and FIPED were assigned as group A. The fellow eyes of patients enrolled in group A, if the FIPED was not identified based on the above criteria, were considered to be a fellow eye group (group B). We have also included 40 age and sex-matched normal healthy eyes as the control group (group C).

A full ophthalmological examination including best-corrected visual acuity (BCVA), intraocular pressure and fundus examination, as well as EDI-OCT were performed for all patients. In cases in which FIPED was observed in OCT, an optical coherence tomography angiography (OCTA) was additionally obtained.

Overall, in this study we had three groups: A) chronic CSC with FIPED (44 eyes), B) fellow eye group with no FIPED (40 eyes) and C) healthy normal eyes (40 eyes).

### Image acquisition and analysis protocol

Enhanced-depth imaging optical coherence tomography (EDI-OCT) images were obtained using the RTVue XR 100 Avanti instrument (Optovue, Inc., Fremont, CA, USA). Patients were positioned appropriately and equally spaced OCT B-scans at 8mm x 12mm raster patterns were captured. The scan passing through the fovea was selected for image analysis. Subfoveal choroidal thickness (SFCT), defined as the distance between the outermost border of Bruch’s membrane-RPE complex and the innermost border of the choroidoscleral junction in the subfoveal region, was measured manually with the aid of built‑in calipers in OCT software. All manual segmentations including delineation of choroidal boundaries and FIPED measurements were conducted by a skilled grader (RMB) and rechecked by another independent grader (AM). In case of any dispute, the outlines were segmented by consensus. As choroidal structures exhibit diurnal variations, so all EDI-OCT scans were performed between 9:00 and 12:00.

In cases in which FIPED was detected in EDI-OCT, a detailed analysis was performed by two unmasked graders (AM, RMB) and the following parameters were reported: height, width, and the reflectivity of FIPED as well as intraretinal fluid (IRF), and subretinal fluid (SRF). The reflectivity of FIPED was reported as hypo- or hyperreflective comparing to the reflectivity of the inner plexiform layer (IPL).

The presence of CNV was determined using the AngioVue (Optovue, Inc., Fremont, CA, USA) OCTA system by investigating the outer retina and choriocapillaris slabs. All automated segmentations of OCTA devices were reassessed by manual movements of segmentations to confirm the presence of CNV under FIPEDs.

Eyes were categorized as vascularized FIPED (vFIPED) if CNV could be exhibited beneath the FIPED, otherwise, it was called avascular FIPED (aFIPED). The automatic measurement of choriocapillaris flow (CC flow) was also documented in each group.

### Evaluation of choroidal area (CA) and choroidal vascular index (CVI) by binarization technique

Discerned independently by unmask graders, distinguishable choroidal images on EDI-OCT were selected to be reviewed for calculation of CVI by Sonoda’s Method [8]. In EDI-OCT images, the upper margin of the region of interest (ROI) was RPE and the lower margin was the choroidoscleral border. The nasal margin was the edge of the optic nerve head and the temporal margin was 8 mm temporal from the edge of the optic nerve head. The distance was determined by the auto-adjust function embedded in the OCT instrument. The binarization of the choroidal area in the OCT image was done by a modified Niblack method as reported [8]. Briefly, the OCT image was analyzed by (FIJI [an expanded version of ImageJ software], version 1.51h; National Institutes of Health, Bethesda, Maryland) available at http://imagej.nih.gov/Fiji/). An ROI was selected and set by the ROI manager in the OCT image. Then, 3 choroidal vessels with lumens larger than 100 *μ*m were randomly selected by the oval selection tool of the toolbar, and the average reflectivity of these areas was determined by the software. The average brightness was set as the minimum value to minimize the noise in the OCT image. Then, the image was converted to 8 bits and adjusted by the auto local threshold of Niblack. The binarized image was reconverted to an RGB image, and the luminal area was determined using the color threshold tool. The light pixels were defined as the choroidal stroma or interstitial area and the dark pixels were defined as the luminal area. The total choroidal area (TCA), luminal area (LA), and stromal area (SA) were automatically calculated. Herein, we refer to the ratio of LA to TCA as the choroidal vascular index (CVI), and the ratio of SA to TCA as the stromal index (SI).

#### Inter-rater agreement

To evaluate inter-rater reliability for SFCT measurement, PED length and height computation, and ultimately CVI measurement, the absolute agreement model of the inter-class correlation coefficient (ICC) was employed on twenty EDI-OCT images, which were initially segmented by two independent graders. A correlation value of 0.81–1.00 indicates good agreement.

### Statistical analysis

To describe data, we used mean, standard deviation, median, range, frequency, and percentage. To compare the results between groups when considering the probable correlation of the eyes, we used Generalized Estimating Equation (GEE). Multiple comparisons are considered by the Sidak method. Multivariate logistic regression analyses were made to determine the clinical factors associated with the occurrence of vFIPED, with a GEE model. A P-value of less than 0.05 is considered statistically significant. All statistical analysis was performed by SPSS (IBM Corp. Released 2017. IBM SPSS Statistics for Windows, Version 25.0. Armonk, NY: IBM Corp).

## Results

### Characteristics of eyes in three groups (FIPEDs, fellow eyes and healthy controls)

The mean age was 50.6 ± 11.2 and 50.2 ± 9 years old in the patients and healthy subjects, respectively (p >0.99). The mean BCVA was 0.5 ± 0.38, 0.14 ± 0.27 and 0.01 ± 0.03 LogMAR in the group A, B and C, respectively (p<0.001). The baseline demographics of the enrolled patients are given in Table 1.

**Table 1:** Demographics of patients with chronic CSC in different groups.

		Total	Group			P†	FIPED		P†
			FIPED	Fellow eye	Control		Vascularized	Avascular	
**Age** (mean ± SD)		50.2 ± 10.4	50.6 ± 11.2	49.9 ± 11.2	50.2 ± 9	>0.99	55.5 ± 10.7	47.7 ± 10.7	0.016
Median (Range)		48 (29 to 74)	48 (29 to 74)	48 (29 to 74)	50.5 (33 to 68)		55 (40 to 74)	47.5 (29 to 71)	
**Refraction (SE)** (mean ± SD)		-0.33 ± 1	-0.39 ± 1.06	-0.34 ± 1.14	-0.27 ± 0.83	0.924	-0.12 ± 1.26	-0.55 ± 0.91	0.292
Median (Range)		-0.15 (-4.5 to 1.75)	-0.25 (-4 to 1.75)	0 (-4.5 to 1.5)	-0.25 (-2 to 1.25)		0 (-4 to 1)	-0.5 (-2.5 to 1.75)	
**BCVA** (mean ± SD) logMAR		0.21 ± 0.34	0.5 ± 0.38	0.14 ± 0.27	0.01 ± 0.03	<0.001	0.54 ± 0.38	0.48 ± 0.39	0.892
Median (Range)		0 (0 to 1.3)	0.52 (0 to 1.3)	0 (0 to 1)	0 (0 to 0.1)		0.4 (0.1 to 1.3)	0.52 (0 to 1)	
**Sex**	M	102 (82.3%)	37 (84.1%)	33 (82.5%)	32 (80.0%)	0.748	11 (78.6%)	26 (86.7%)	0.515
	F	22 (17.7%)	7 (15.9%)	7 (17.5%)	8(20.0%)		3 (21.4%)	4 (13.3%)	
**Eye**	OD	62 (50.0%)	20 (45.5%)	22 (55.0%)	20 (50.0%)	0.662	6 (42.9%)	14 (46.7%)	0.813
	OS	62 (50.0%)	24 (54.5%)	18 (45.0%)	20 (50.0%)		8 (57.1%)	16 (53.3%)	

BCVA: Best-corrected visual acuity, SE: Spherical equivalent, FIPED: Flat irregular pigment epithelial detachment.

†Based on generalized estimating equation (GEE).

Table 2 shows acceptable inter-rater agreements in assessment of SFCT, PED length and height as well as CVI measurement.

**Table 2:** Inter-rater reliability assessment of measured parameters.

	Interclass correlation coefficient (ICC)	95% confidence interval (CI)
CVI	0.969	0.918–0.988
SFCT	0.988	0.970–0.995
PED height	0.992	0.979–0.997
PED width	0.990	0.975–0.996

CVI: Choroidal vascular index, SFCT: Subfoveal choroidal thickness, PED: Pigment epithelial detachment.

Quantitative EDI-OCT parameters (TCA, CVI, SA, and SFCT), as well as CC flow in age-matched healthy subjects and patients, are shown in Table 3. Mean SFCT for FIPED eyes was 428± 144 mm, which was significantly higher than fellow eyes (394±122) and healthy subjects (350±91) (p = 0.005 and p = 0.006, respectively). A significantly higher TCA was observed in the FIPED group compared with the fellow eye group (p < 0.001), while such a significant difference was not detected between the FIPED group and the control eye group (p = 0.066). The CVI was not significantly different between the three groups. (p = 0.906) The measured CC flow was also significantly lower in the FIPED group compared to the fellow eye and control eye (p<0.001 for both)

**Table 3:** Choroidal structure evaluation in three different groups.

	Total	Group			P†	Multiple comparison‡		
		FIPED	Fellow eye	Control		P1	P2	P3
CVI	75.32 ± 4.17	75.47 ± 4.45	75.4 ± 4.58	75.06 ± 3.45	0.906	-	-	-
SI	24.68 ± 4.17	24.53 ± 4.45	24.6 ± 4.58	24.94 ± 3.45	0.906	-	-	-
TCA	2.96 ± 0.88	3.17 ± 0.97	2.9 ± 0.94	2.8 ± 0.67	0.003	0.001	0.066	0.682
SFCT	392.37 ± 125.8	428.77 ± 144.53	394.58 ± 122.95	350.22 ± 91.7	0.005	0.005	0.006	0.113
CC flow	4.45 ± 0.52	4.09 ± 0.54	4.71 ± 0.5	4.59 ± 0.26	<0.001	<0.001	<0.001	0.202

FIPED: Flat irregular pigment epithelial detachment, CVI: Choroidal vascular index, SI: Stromal index, TCA: Total choroidal area, SFCT: Subfoveal choroidal index, CC flow: Choriocapillaries flow.

†Based on generalized estimating equation (GEE).

‡ Based on Sidak method.

P1: PED vs Fellow eye, P2:PED vs Control, P3:Fellow eye vs Control.

### Characteristics of eyes with vascularized and avascular FIPEDs

Overall, CNV was discovered in 14 eyes of group A (31.81%). A hyperintense flow signal between BM and RPE in flow overlay B scans of OCT-A and a well-delineated CNV network on en-face OCT-A was detected in all these 14 eyes. There was a correlation between age and CNV occurrence based on univariate analysis (p = 0.016). However, such correlation was not observed between gender and CNV incidence (p = 0.515). The mean BCVA (LogMAR) was not significantly different in eyes with vFIPED and the eyes with aFIPED (0.54 ± 0.38 vs. 0.48 ± 0.39, p = 0.892) (Table 1).

The evaluated imaging parameters between the aFIPEDs and vFIPEDs subgroups are reported in Table 4. The mean value of PED height in eyes with vFIPED was higher than in eyes with aFIPED, nevertheless, this difference was not statistically significant (p = 0.136). The width of FIPED was not remarkably different between these subgroups (p = 0.986).

**Table 4:** Comparison between avascular and vascularized FIPEDs in chronic CSC.

Quantitative		Total	FIPED		Univariate analysis				Multivariate analysis			
variable			Vascularized	Avascular	OR	95% CI		P†	OR	95% CI		P†
CVI		75.47 ± 4.45	77.37 ± 4.12	74.58 ± 4.38	1.12	1.01	1.30	**0.025**	1.41	1.05	1.89	**0.020**
TCA		3.17 ± 0.97	2.75 ± 0.53	3.36 ± 1.07	0.41	0.20	0.85	**0.016**	4.77	0.52	43.76	0.167
SFCT		428.77 ± 144.53	349.79 ± 62.13	465.63 ± 157.56	0.98	0.98	0.99	**0.004**	0.97	0.95	0.99	**0.003**
CC Flow		4.09 ± 0.54	4.16 ± 0.65	4.06 ± 0.48	1.37	0.36	5.17	0.639				
PED height		70.91 ± 31.87	84.85 ± 29.79	64.87 ± 31.28	1.01	0.99	1.04	0.136				
PED width		1312.37 ± 769.01	1313.54 ± 616.21	1311.87 ± 836.27	1.00	0.99	1.01	0.974				
Qualitative		Total	FIPED		OR	95% CI		P†				
variable			Vascularized	Avascular		Lower	Upper					
PED reflectivity	hyper + iso	21 (47.7%)	10 (71.4%)	11 (36.7%)	4.15	1.04	16.65	**0.044**	7.32	0.72	74.43	0.092
	hypo	23 (52.3%)	4 (28.6%)	19 (63.3%)	ref							
IRF	no	40 (90.9%)	11 (78.6%)	29 (96.7%)	ref							
	yes	4 (9.1%)	3 (21.4%)	1 (3.3%)	7.60	0.71	81.23	0.094				
SRF	no	0 (0%)	0 (100%)	0 (0.0%)	ref							
	yes	44 (100%)	14 100%)	30 (100.0%)	NA	-	-					

FIPED: Flat irregular pigment epithelial detachment, CVI: Choroidal vascular index, SI: Stromal index, TCA: Total choroidal area, SFCT: Subfoveal choroidal thickness, CC flow: Choriocapilaries flow, IRF: Intra retinal fluid, SRF: Subretinal fluid, AUC: Area under the Curve.

†Based on binary logistic analysis. A generalized estimating equation (GEE) was used to adjust for intereye correlation of the same patients.

Based on the univariant analysis, the mean SFCT (p = 0.004) and TCA (p = 0.016) values were lower in eyes with vFIPED compared to those with aFIPED. Mean CVI was significantly higher in eyes with vFIPED in comparison to eyes with aFIPED. (p = 0.025).

Of note, FIPEDs with iso-hyper internal reflectivity were significantly associated with the presence of CNV beneath the FIPED (P = 0.044).

Among 14 patients with vFIPED, 3 (21%) and 14 (100%) eyes showed to have variable amounts of intraretinal and subretinal fluid, respectively at the time of OCTA acquisition. In eyes with aFIPED, IRF and SRF were detected in 1 (3.3%) and 30 (100%) eyes, respectively. No association was found between the presence of SRF or IRF in B-scan and detection of CNV in OCTA.

**Fig 1:**
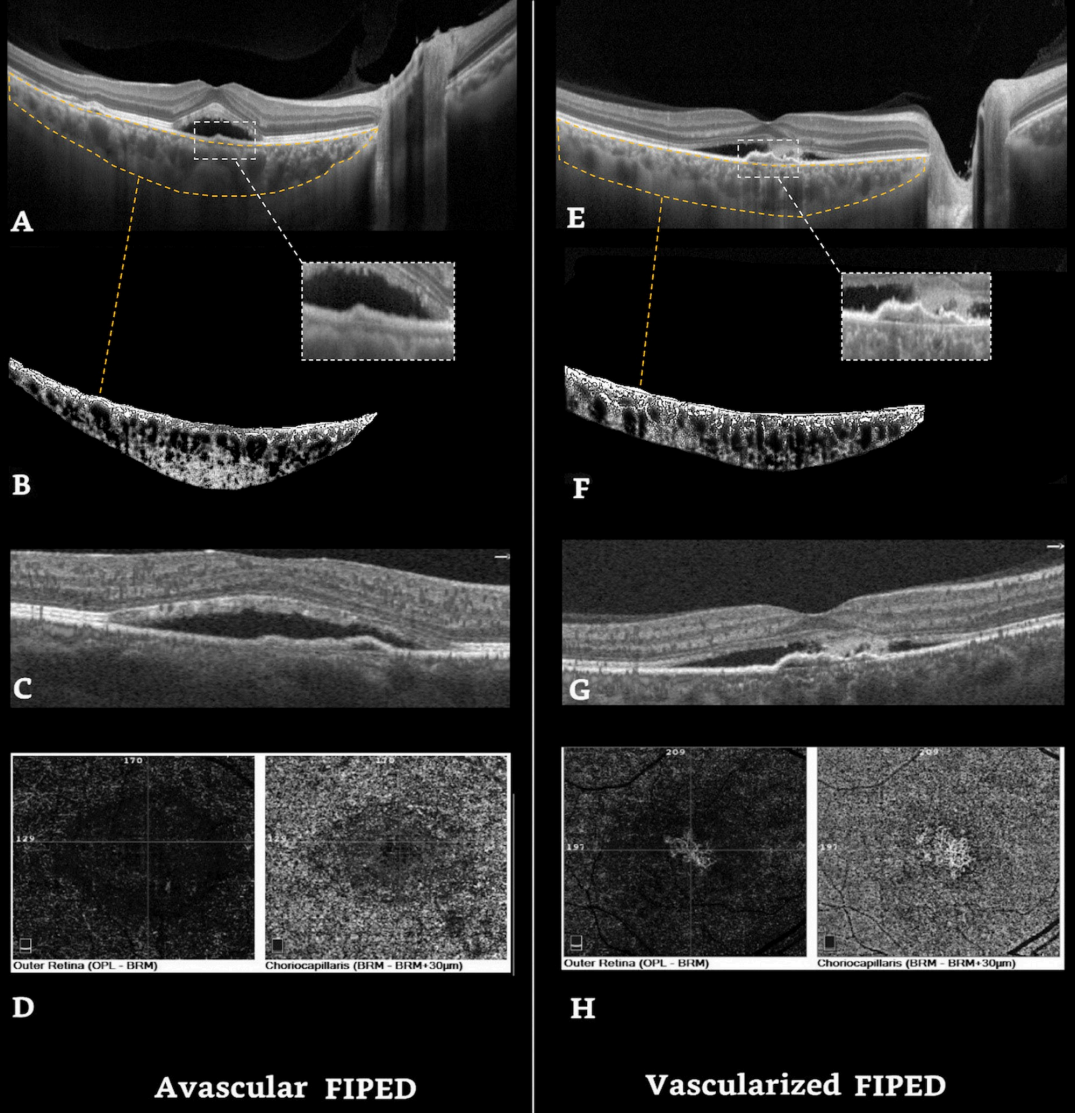
Right eye EDI-OCT and OCTA images of chronic CSC in a patient with avascular FIPED (A-D) and a patient with vascularized FIPED (E-H). A and E show corresponding EDI-OCT scanning foveal area, B and F show corresponding choroidal vasculature overlay performed with the Sonoda’s technique. C and G show the flow overlay of the OCTA B-scan in the foveal area, G illustrates the flow in the FIPED area. D and H show OCTA enface images, outer retina, and choriocapillaris slabs. H reveals the CNV in both slabs in vascularized FIPED. FIPED: Flat irregular pigment epithelial detachment.

Age, CVI, choroidal area, SFCT, and PED reflectivity were analyzed in a multivariate regression analysis model, and only CVI and SFCT were found to be independent features in association with the occurrence of vFIPED. Higher CVI had a positive correlation with the finding of CNV beneath the FIPED (OR: 1.41, CI95%: 1.05–1.89; p = 0.020), and SFCT showed to have a negative correlation (OR: 0.97, CI95%: 0.95–0.99; p = 0.003) (Table 4) (Fig 1).

In subgroup analysis for comparison of choroidal biomarkers between two subtypes of FIPEDs versus fellow eyes and controls, CVI in the aFIPED eyes was not significantly different from fellow eyes and controls, separately (74.58 ± 4.38 vs 74.43 ± 4.68 vs75.06 ± 3.45 respectively and overall p-value = 0.809). On the other hand, CVI of vFIPED was larger than fellow eyes and controls although this difference was not statistically significant (77.37 ± 4.12 vs 77.13 ± 3.96 vs 75.06 ± 3.45, respectively and overall p-value = 0.103) (S1 and S2 Tables).

## Discussion

In the current research, eyes with FIPED had thicker choroids, wider choroidal areas, and lower CC flow relative to either fellow eyes with uncomplicated pachychoroid or eyes of healthy subjects, but the CVI discrepancy was not statistically significant between these groups. Moreover, in 44 eyes with chronic CSC and FIPED, OCTA detected vFIPED in 14 eyes (31.8%) which is compatible with the diagnosis of pachychoroid neovasculopathy (PNV). Among different choroidal factors, CVI and SFCT were found to be independent features to be associated with the occurrence of vFIPED.

In published studies using OCTA, the rate of CNV detection within FIPEDs has high variability, probably because of different patient selection criteria (i.e., inhomogeneous CSCR cohorts) or the different OCTA settings [5]. Although OCTA detection rate for type 1 CNV under shallow irregular PEDs was relatively high at 95% in a study conducted by Dansingani et al, most previous studies reported a CNV detection rate between 30 to 35.6% of the eyes [7,9–12]. Lupidi et al showed that the rate of detecting CNV on en face scans increases from 31% in the rest position to 37% during the handgrip test [5].

Choroidal thickness measurements vary among different studies, as this can be affected by factors such as patient’s ethnicity, age, refractive error, and axial length, the light source used in the OCT device, and software used to measure [13,14]. Although it has been shown that the Iranian population has a thicker choroid ranging from 329 to 363 μm compared to some other ethnicities [13,14]. The current study showed that patients with FIPED had a higher choroidal thickness compared to the fellow eye and also normal eyes.

Although choroidal thickness (CT) is considered an important indicator in the spectrum of pachychoroid and its related complications, it does not distinguish vascular from stromal components [15–17]. Therefore, since 2014, the focus of studies switched to find a method to analyze choroidal structure with the better distinction of lumen and stroma. Sonoda et al. first proposed such a method [8], and then Agrawal et al. modified Sonoda’s technique and defined CVI as the choroidal lumen divided by the total choroidal region [18]. According to previous investigations, CVI showed lesser changeability and was impacted by less physiologic elements rather than CT, demonstrating to be a generally steady biomarker for investigating the choroidal changes [19,20].

Subsequent studies comparing the outputs of Agrawal’s and Sonoda’s methods showed that these two methods have a relatively low agreement [21]. Overall, Agrawal’s method measures, the average CVI higher than Sonoda’s method [20,21]. Therefore, it is important to note exactly what algorithm has been used in various studies to measure CVI.

In the present study, the mean CVI of normal individuals measured 75.06 ± 3.45 using Sonoda’s technique. As with choroidal thickness, the mean CVI in normal individuals in the current study appears to be higher than that measured in other studies [22,23]. Agarwal et al, measured mean subfoveal CVI (1500 μm wide) 65.61 ± 2.33, in a study of 345 healthy subjects with the same ethnicity [19]. This discrepancy may be related to factors such as ethnicity, the width of the subfoveal region used to assess CVI, and the method of measurement.

Of note, for a more accurate estimation of CVI, we extended our measurement from the optic nerve to the temporal side of the fovea at 8mm. Although Agrawal and his colleagues showed that in normal individuals, subfoveal CVI can well represent CVI of the entire macular region [19], this statement may not be true in pachychoroid disease or ethnicities with the thicker choroid.

The mean CVI of patients with FIPED in our study did not show a significant difference compared to the opposite eye and normal individuals. It seems that CVI is not a strong predictor for the presence of FIPED.

**Fig 2:**
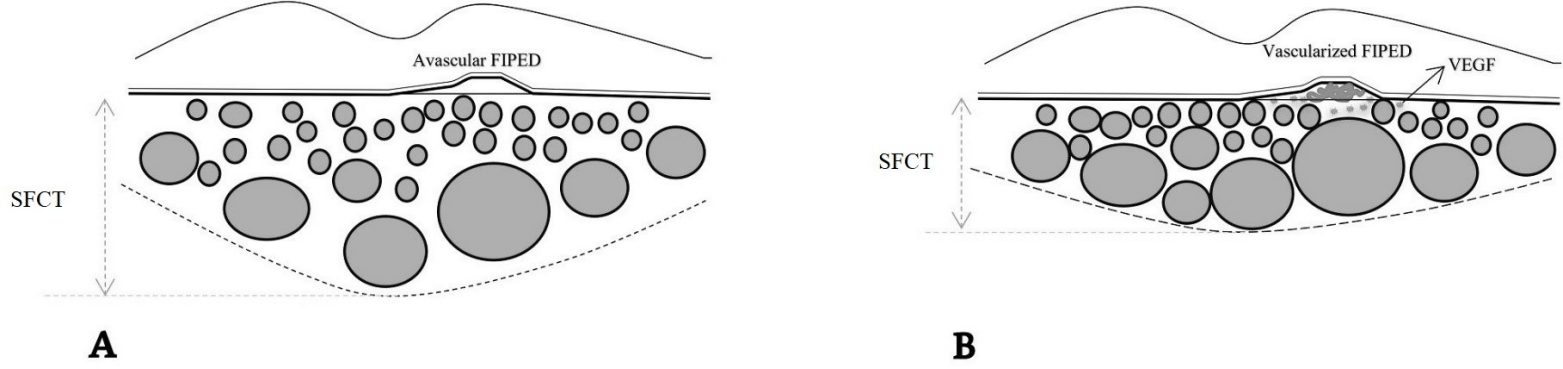
Choroidal features in patients with aFIPED (A) showed greater choroidal thickness and choroidal area, and lower CVI in comparison to vFIPED (B). Dilated pachyvessels impose a mechanical pressure on choriocapillaris which subsequently causes attenuation of the inner choroid, VEGF production (stars), and new vessel formation. SFCT: Subfoveal choroidal thickness, FIPED: Flat irregular pigment epithelial detachment.

Current investigation showed that patients with vFIPED have older age, lower choroidal thickness and choroidal area, and higher CVI in comparison to patients with aFIPED. Additionally, the material within the PED of a vFIPED was noticeably hyper or iso-reflective rather than hypo-reflective. In a multivariate analysis including these parameters, CVI and choroidal thickness were found to be independent factors to predict vFIPED (Fig 2).

Similarly, Guo et al. evaluated characteristics of vFIPED in comparison to aFIPED and found lower choroidal thickness in the former [24]. Kim et al. also evaluated choroidal thickness and CVI in four subgroups of patients with CSC including acute CSC, chronic CSC without PED, chronic CSC with FIPED, and chronic CSC with FIPED and CNV. They found lower choroidal thickness (387 ± 26 μm) and CVI (67.18 ± 6.73) in eyes with FIPED and CNV in comparison to other subgroups. They proposed that the changes in the CVI are particularly relevant to the presence of CNV, rather than reflecting clinical characteristics of CSC [25].

On the contrary, our measured CVI was higher in vFIPED compared to aFIPED. This might be attributed to different stages and duration of disease activity with various levels of choroidal ischemia which can affect CVI or may be related to different areas of measurement.

The lower total choroidal area and higher CVI in eyes with vFIPED in comparison to aFIPED may indicate different pathogenic mechanisms involved in each entity. The pathogenesis of CNV in the pachychoroid spectrum is not fully elucidated. It has been hypothesized that dilated pachyvessels induce a mechanical pressure on choriocapillaris which subsequently lead to obliteration and atrophy of this layer which in turn upgrade VEGF production and promote new vessel formation [6,26–28]. Our results are in accordance with this theory and imply that in eyes with vFIPED, the choroid is composed of more dilated and congested vessels with attenuated stromal and choriocapillaris area. Fung et al speculated that long-standing PED in patients with pachychoroid could result in a split in Bruch’s membrane and subsequent CNV formation [29]. Our results additionally suggested that choroidal vascular changes are also associated with the development of CNV. To verify this hypothesis, further studies need to be conducted.

The association of PED height and the presence of hyperreflectivity of material under PED with the presence of CNV have been reported by other studies as well which is consistent with the current study [7,30,31].

Numerous theories of CSC pathophysiology have focused on multifocal choroidal vascular hyperpermeability since the debut of ICG angiography. Widefield ICG angiography commonly reveals venous dilation and choroid vascular filling delays [32,33]. The occurrence of intervortex venous anastomoses in CSC provides evidence to the theory of venous outflow problems. Flow anomalies may influence one (or more) of the vortex veins networks, resulting in congestion and increased venous pressure [34–37]. Pigment epitheliopathy was connected to the thicker choroid and increased ocular perfusion pressure in CSC patients’ eyes [38]. These concerns could have influenced the development of FIPED or the shift from aFIPED to vFIPED. To put this concept to the test, we propose a long-term investigation on CSC patients utilizing widefield ICG imaging to investigate the relationship between FIPED, intervortex venous anastomosis, and choroidal hyperpermeability.

Our study has some limitations such as the relatively small sample size and retrospective design. Also, given the cross-sectional design of the current study, we could not assess longitudinal changes of FIPEDs e.g. elongation of a FIPED overtime, as Alex, et al showed that lateral elongation may be considered as a precursor lesion for Polypoidal Choroidal Vasculopathy and as a novel OCT biomarker for the disease activity [39]. The study was conducted in a tertiary center, which may lead to the recruitment of more chronic cases, leading to selection bias. The duration of symptoms was not specified which might affect the morphologic features of the neovascular membrane. Another limitation of the current study was that we did not assess any correlation of FIPED type with leakage points in dye-based angiographies (FFA/ICGA) which could be helpful to precisely differentiate aFIPED from vFIPED and also to associate sites of choroidal vascular hyperpermeability and FIPEDs.

Image processing methods for the calculation of CVI have various drawbacks that should be addressed here. Our validation of CVI’s actual applicability in the general population is still pending. The retinal blood vessels may cast shadows on OCT imaging of the posterior segment, making it difficult to see the choroid. Many OCT machines include an algorithm to "flatten" the generated image so that it fits better on the report sheets. This distorts the actual anatomy, which may have an impact on CVI computation. The dark and white pixels in the binarized image were thought to represent the vascular and stroma areas, which may or may not be correct [40].

The advantage of our study is that we assessed choroidal vascular changes in larger and more extended areas over the macula compared to other studies.

In conclusion, although CVI in contrast to choroidal parameters such as TCA and SFCT was not associated with the presence of FIPED in patients with chronic CSC, it seems that measurement of CVI along with SFCT may help to differentiate aFIPED from vFIPED in patients with CSC. Further longitudinal studies would be required to confirm the clinical significance of these findings.

## Supporting information

S1 Table Choroidal structure evaluation in eyes with aFIPED, fellow eyes, and healthy controls. (DOCX)

S2 Table Choroidal structure evaluation in eyes with vFIPED, fellow eyes, and healthy controls. (DOCX)
