# Supplementary material for: Choroidal features in flat irregular pigment epithelial detachment associated with Chronic central serous chorioretinopathy: Avascular versus vascularized
Source: PLoS One. 2021 Sep 23;16(9):e0257763. doi: 10.1371/journal.pone.0257763 (PMC8459941; doi:10.1371/journal.pone.0257763)
Supplement: S1 Table — (DOCX) [file pone.0257763.s001.docx]

|  | aFIPED | Fellow eye | Control | P value† | Multiple comparison ‡ | | |
| --- | --- | --- | --- | --- | --- | --- | --- |
|  |  |  |  |  | P1 | P2 | P3 |
| CVI | 74.58 ± 4.38 | 74.43 ± 4.68 | 75.06 ± 3.45 | 0.809 | >0.990 | 0.902 | 0.940 |
| SI | 25.42 ± 4.38 | 25.57 ± 4.68 | 24.94 ± 3.45 | 0.809 | >0.990 | 0.902 | 0.940 |
| TCA | 3.36 ± 1.07 | 3.11 ± 1.06 | 2.80 ± 0.67 | **0.002** | **0.004** | **0.046** | 0.636 |
| SFCT | 465 ± 157 | 437 ± 130 | 350 ± 91 | **0.002** | **0.037** | **0.002** | **0.031** |
| ccflow | 4.06 ± 0.48 | 4.75 ± 0.58 | 4.59 ± 0.26 | **<0.001** | **<0.001** | **<0.001** | 0.448 |

S1 Table: Choroidal Structure Evaluation in eyes with aFIPED, fellow eyes, and healthy controls.

aFIPED: avascular Flat irregular pigment epithelial detachment, CVI: Choroidal vascular index, SI: Stromal index, TCA: Total choroidal area, SFCT: Subfoveal choroidal index, CC flow: Choriocapillaries flow

†Based on generalized estimating equation (GEE).

‡ Based on Sidak method.

P1: PED vs Fellow eye, P2:PED vs Control, P3:Fellow eye vs Control.
