# Supplementary material for: Choroidal features in flat irregular pigment epithelial detachment associated with Chronic central serous chorioretinopathy: Avascular versus vascularized
Source: PLoS One. 2021 Sep 23;16(9):e0257763. doi: 10.1371/journal.pone.0257763 (PMC8459941; doi:10.1371/journal.pone.0257763)
Supplement: S2 Table — (DOCX) [file pone.0257763.s002.docx]

S2 Table: Choroidal Structure Evaluation in eyes with vFIPED, fellow eyes, and healthy controls.

|  | vPED | Fellow eye | Control | P value† | Multiple comparison ‡ | | |
| --- | --- | --- | --- | --- | --- | --- | --- |
|  |  |  |  |  | P1 | P2 | P3 |
| CVI | 77.37 ± 4.12 | 77.13 ± 3.96 | 75.06 ± 3.45 | 0.103 | 0.995 | 0.148 | 0.199 |
| SI | 22.63 ± 4.12 | 22.87 ± 3.96 | 24.94 ± 3.45 | 0.103 | 0.995 | 0.148 | 0.199 |
| TCA | 2.75 ± 0.53 | 2.53 ±0.55 | 2.80 ± 0.67 | 0.285 | 0.463 | 0.990 | 0.434 |
| SFCT | 349 ± 62 | 321 ± 62 | 350 ± 91 | 0.342 | 0.468 | >0.990 | 0.569 |
| ccflow | 4.16 ± 0.65 | 4.62 ± 0.30 | 4.59 ± 0.26 | 0.004 | 0.003 | 0.042 | 0.972 |

vFIPED: vascularized Flat irregular pigment epithelial detachment, CVI: Choroidal vascular index, SI: Stromal index, TCA: Total choroidal area, SFCT: Subfoveal choroidal index, CC flow: Choriocapillaries flow

†Based on generalized estimating equation (GEE).

‡ Based on Sidak method.

P1: PED vs Fellow eye, P2:PED vs Control, P3:Fellow eye vs Control.
